# Prostate cancer in young men represents a distinct clinical phenotype: gene expression signature to predict early metastases

**DOI:** 10.20517/jtgg.2021.01

**Published:** 2021-03-09

**Authors:** Yuan C. Ding, Huiqing Wu, Elai Davicioni, R. Jeffrey Karnes, Eric A. Klein, Robert B. Den, Linda Steele, Susan L. Neuhausen

**Affiliations:** 1 Department of Population Sciences, Beckman Research Institute of City of Hope, Duarte, California, CA 91010, USA.; 2 Department of Pathology, City of Hope Medical Center, Duarte, California, CA 91010, USA.; 3 GenomeDX Biosciences, Vancouver, British Columbia V6B 1B8, Canada.; 4 Department of Urology, Mayo Clinic, Rochester, Minnesota, MN 55905, USA.; 5 Glickman Urological and Kidney Institute, Cleveland Clinic, Ohio, OH 44195, USA.; 6 Department of Radiation Oncology, Thomas Jefferson University, Philadelphia, Pennsylvania, PA 19044, USA.

**Keywords:** Differentially expressed gene, immune cell enrichment, metastasis, prostate cancer, tissue microenvironment, age, prediction, patient stratification, clinical phenotype

## Abstract

**Aim:**

Several genomic signatures are available to predict Prostate Cancer (CaP) outcomes based on gene expression in prostate tissue. However, no signature was tailored to predict aggressive CaP in younger men. We attempted to develop a gene signature to predict the development of metastatic CaP in young men.

**Methods:**

We measured genome-wide gene expression for 119 tumor and matched benign tissues from prostatectomies of men diagnosed at ≤ 50 years and > 70 years and identified age-related differentially expressed genes (DEGs) for tissue type and Gleason score. Age-related DEGs were selected using the improved Prediction Analysis of Microarray method (iPAM) to construct and validate a classifier to predict metastasis using gene expression data from 1,232 prostatectomies. Accuracy in predicting early metastasis was quantified by the area under the curve (AUC) of receiver operating characteristic (ROC), and abundance of immune cells in the tissue microenvironment was estimated using gene expression data.

**Results:**

Thirty-six age-related DEGs were selected for the iPAM classifier. The AUC of five-year survival ROC for the iPAM classifier was 0.87 (95%CI: 0.78–0.94) in young (≤ 55 years), 0.82 (95%CI: 0.76–0.88) in middle-aged (56–70 years), and 0.69 (95%CI: 0.55–0.69) in old (> 70 years) patients. Metastasis-associated immune responses in the tumor microenvironment were more pronounced in young and middle-aged patients than in old ones, potentially explaining the difference in accuracy of prediction among the groups.

**Conclusion:**

We developed a genomic classifier with high precision to predict early metastasis for younger CaP patients and identified age-related differences in immune response to metastasis development.

## INTRODUCTION

Prostate cancer (CaP) is primarily a disease of older men; only 9.2% of men develop CaP under age 55 years^[1]^. Although the overall incidence of CaP is decreasing in the United States^[1]^, the incidence is increasing in younger (≤ 55 years) men compared to older men (> 70 years)^[2**–**4]^. Due to a longer life expectancy, younger men with localized CaP are more likely to receive radical prostatectomy (RP) treatment than older men^[5,6]^. A recent long-term follow-up study demonstrated that only those patients harboring lethal CaP and having a long-life expectancy benefited from RP treatment^[7]^. Over the past decade, several genomic signatures have been developed to prognostic signature was tailored to predict expression in prostatectomy or biopsy tissues^[8]^. However, no prognostic signature was tailored to predict aggressive CaP in men younger than age 55 years. Converging data from clinical and molecular genetic studies provide strong evidence that CaP in young men represents a distinct clinical phenotype with underlying biological differences compared to older men^[9**–**13]^. We hypothesized that age-related differences in tumor biology have implications for prognosis of early-onset CaPs. To test this hypothesis, we selected tumor and matched benign prostatic tissue samples from men diagnosed with CaP at younger (≤ 50 years) and older (71–75 years) ages with low (6), intermediate (7), and high (8–10) Gleason scores. We identified age-related differentially expressed genes (DEGs) by comparing sample type (tumor versus matched benign) and Gleason scores (low *vs.* high). Then we developed a genomic classifier using gene expression of age-related DEGs and tested the classifier for accurate identification of young patients with aggressive CaP as defined by metastasis within five years of RP.

## METHODS

### Patient characteristics, mRNA profiling, and identification of age-related DEGs

This study was approved by the City of Hope (COH) Institutional Review Board (IRB07244). Patients with CaP and treated with RP between 1998 and 2013 at COH National Medical Center were selected based on age at diagnosis and tissue availability [Table 1]. A total of 61 men diagnosed between the ages of 71 and 75 years (old) and 58 men diagnosed between the ages of 38 and 50 years (young) were used to identify age-related DEGs for developing a gene expression classifier to predict metastasis following RP. Older cases were matched to younger cases for cancer stage and Gleason score. Tissue processing and mRNA profiling were performed as described^[9]^. Follow-up data were abstracted from medical records and the COH cancer registry. Age-related DEGs were identified from expression data using a mixed linear model implemented in limma R^[14]^ [Supplementary methods].

### iPAM classifier development and validation

**Figure 1.**
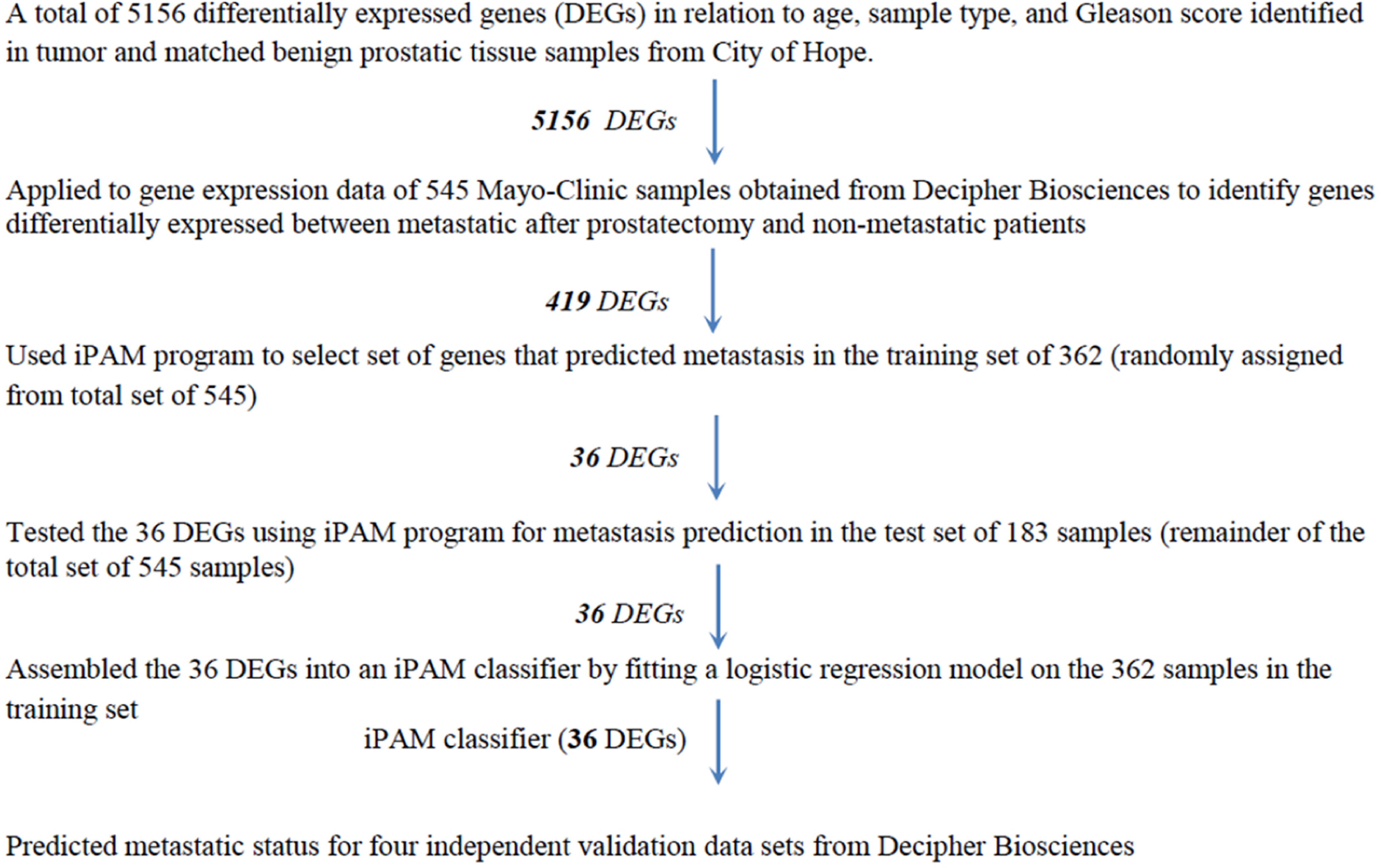
Study design for developing the iPAM classifier.

Gene expression data (46,050 genes and 1,232 patients from RP) from the Decipher Genomic Resource Information Database (GRID, Decipher Biosciences, San Diego, CA) [Supplementary Table 1] were used to develop and validate a new genomic classifier. The study design is shown in Figure 1. Gene expression data for the age-related DEGs were extracted from the Mayo Clinic (MC I) discovery cohort^[15]^. A two-sample *t*-test selected DEGs between patients with and without metastasis. After DEG determination, patients were randomly assigned into training (*n* = 362) and test (*n* = 183) datasets. An improved Prediction Analysis of Microarray (iPAM) method^[16**–**18]^ removed DEGs irrelevant to metastasis prediction based on minimizing the 10-fold cross-validated error rate using the Adaptive Hierarchically Penalized Nearest Shrunken Centroid algorithm^[17]^. These iPAM-selected DEGs were assembled into an iPAM classifier by fitting a logistic regression model on the training set.

Three independent validation data sets [Supplementary Table 1] with follow-up time [the Mayo Clinic II (MC II)^[19]^, the Cleveland Clinic (CC)^[20]^, and the Thomas Jefferson University (TJU)]^[21]^ were used to evaluate the performance of the iPAM classifier by AUC of ROC for censored survival data^[19]^. The 95% confidence interval for AUC of survival ROC was constructed from 1000 bootstrap replications. Based on bimodal distribution of risk scores predicted by the iPAM classifier, two cut points were selected to categorize patients into low-, intermediate-, and high-risk groups. Kaplan-Meier estimator and a log-rank test were used to evaluate the difference in time to metastasis among the risk groups. The conventional AUC of ROC was calculated to measure prediction accuracy for the fourth validation data set from the Memorial Sloan-Kettering Cancer Center (MSKCC), which had no follow-up time but categorical metastasis status for each patient.

### Estimation of cell-type proportion in tissue microenvironment

xCell^[22]^ was used to estimate the abundance of 34 immune cell types for each tissue sample using genome-wide gene expression data. Cell-type proportion in tissue microenvironment estimated by xCell method is a rank-based enrichment score. Non-parametric analysis of variance (ANOVA) (confidence interval and p-values generated by percentile bootstrap) implemented in the “Rallfun-v35” R codes from Dr. Wilcox^[23]^, was used to test median differences in immune score (average abundance of immune cells) between sample groups classified by factors of sample type (tumor, benign), metastasis status (yes, no), and age group (young, middle-aged, old).

## RESULTS

### Identification of age-related DEGs

**Figure 2.**
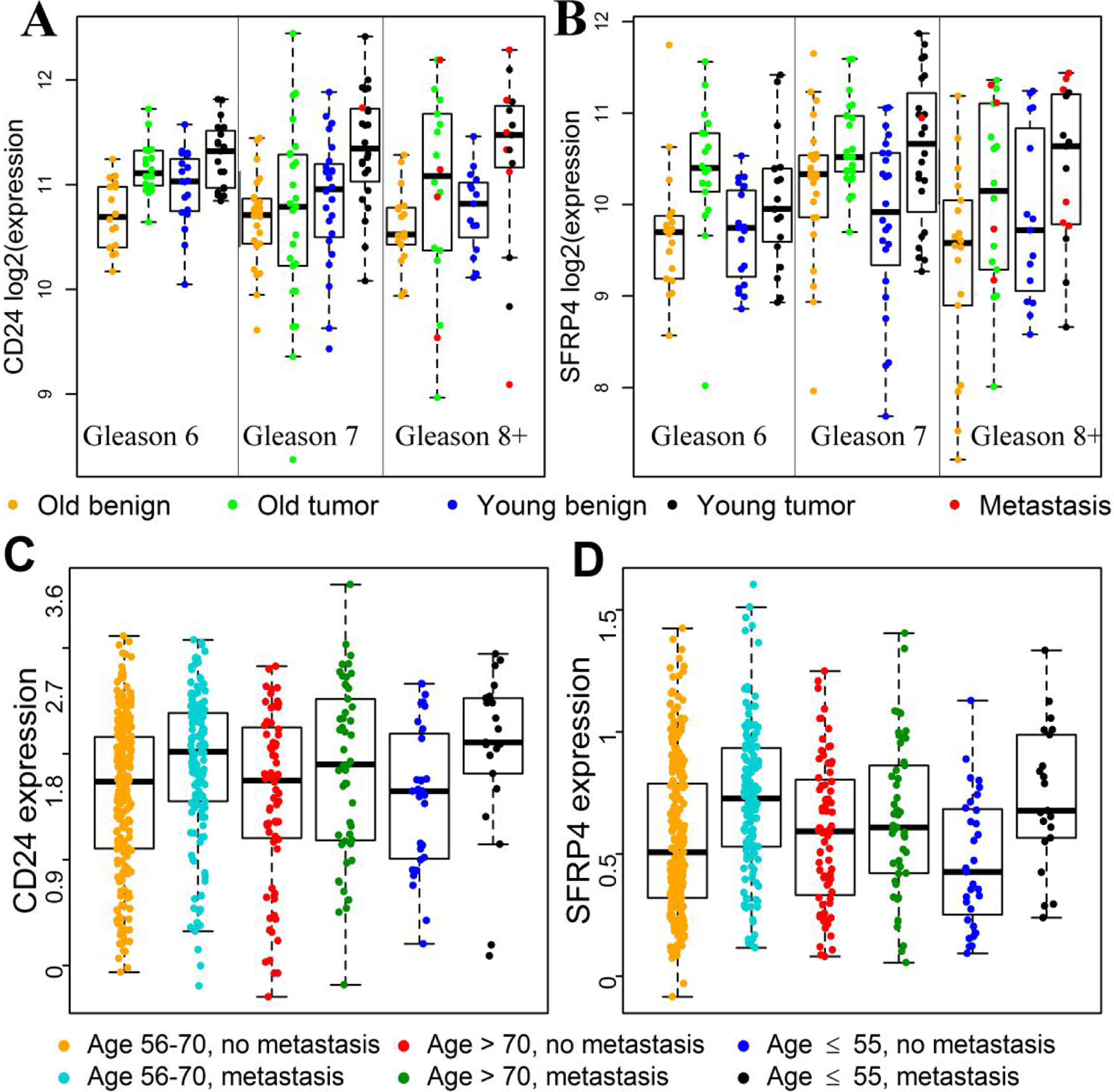
Differential gene expression for *CD24* and *SFRP4* between sample groups classified by patient age, sample type, or metastasis factors. *CD24* and *SFRP4* are 2 of the 36 genes in the iPAM classifier. A and B, Gene expression data from the 119 COH patients were used to generate the box and dot plots; there were significant tumor-*vs.*-matched-benign median expression differences among young patients (≤ 50 years) with Gleason score of 7 for *CD24* (A) and *SFRP4* (B) (black *vs.* blue for young patients, FDR < 0.05); for *CD24* (panel A), median expression level was significantly higher in tumors from young patients than in tumors from old patients with Gleason score of 7 (black *vs.* green, FDR < 0.05). C and D, Gene expression data for 545 patients in the Mayo discovery data set were used to generate plots; among young (≤ 55 years) patients, significantly increased median expression levels for *CD24* (C) and *SFRP4* (D) were observed in patients with metastasis compared to patients without metastasis (black *vs.* blue, FDR < 0.05); however, no significant median expression difference related to metastasis status among patients older than 70 years (red *vs.* green, *P* > 0.2).

We previously identified genes differentially expressed between tumor and matched benign prostatic tissue samples for young men (≤ 45 years) and old men (71–74 years) with Gleason score 7 (3 + 4) CaP^[9]^. Following the same study design, we generated gene expression data for tumor and matched benign prostatic samples from young men (≤ 50 years, *n* = 34) and old men (71–75 years, *n* = 36) with CaP Gleason scores of 6 or 8–10. We identified 5,156 unique DEGs as potential candidate genes for developing the iPAM classifier [Table 2]. Dot plots of gene expression for two DEGs are shown in Figure 2A and Figure 2B as examples. Details on the 5156 DEGs are available upon request.

### iPAM classifier development and performance assessment

**Figure 3.**
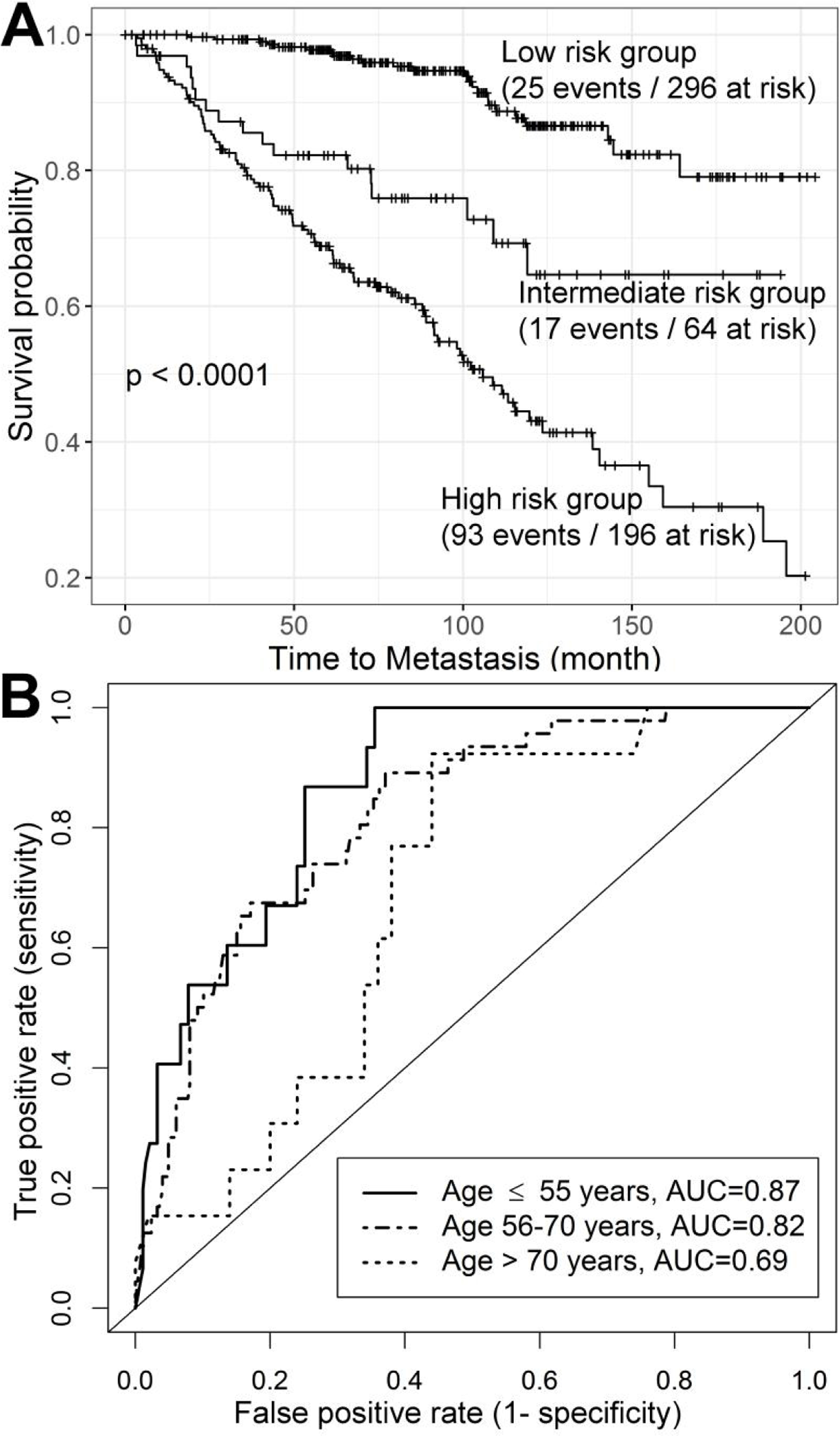
Performance of the iPAM classifier. Combination of patients from Mayo Clinic validation data (*n* = 235), Cleveland Clinic validation data (*n* = 182), and Thomas Jefferson University validation data (*n* = 139) were used to evaluate performance of the iPAM classifier. (A) Three risk groups [low- (iPAM risk score < 0.4), intermediate- (0.4 ≤ iPAM risk score ≤ 0.6), and high-risk (iPAM risk score > 0.6) group] showed highly significant differences in metastasis-free survival (*P* < 0.0001) from the Kaplan-Meier survival analysis. (B) Accuracy in predicting early metastasis (within five years of RP), quantified by AUC of ROC, was higher in young (≤ 55 years) (AUC = 0.87) and middle-aged patients (AUC = 0.82) than in old (> 70 years) patients (AUC = 0.69).

Gene expression data for the 5156 DEGs were extracted from the MC I discovery data set. Of those DEGs, 419 were differentially expressed (false discovery rate [FDR] < 0.05) between patients who did and did not develop metastasis. The iPAM program^[18]^ selected 36 genes [Table 3] of the 419 that predicted metastatic CaP in the training dataset and then generated an AUC of 0.75 for the test data set. We assembled those 36 genes into an iPAM classifier by fitting a logistic regression model on the training samples, and applied the iPAM classifier to four independent validation data sets. The predicted iPAM risk scores for metastasis showed a bimodal distribution with the score range of 0–1, where higher scores represent higher risk of developing metastasis. For the three independent data sets (MC II, CC, TJU, *n* = 556) with follow-up time from the date of RP, 75 of 556 patients (13.5%) developed metastasis within five years after RP (early), 60 patients (10.8%) developed metastasis more than five years after RP (late), and 421 patients had no metastases at last follow-up (mean follow-up time of 91 months). The median iPAM risk scores for the three groups of patients were 0.89, 0.67, and 0.24, respectively. The iPAM risk scores were stratified into high-, intermediate-, and low-risk groups by two cut points of 0.4 and 0.6 determined by the distribution of risk score [Supplementary Figure 1]; the three groups showed highly significant differences in metastasis-free survival (*P* < 0.0001) [Figure 3A]. Of 75 patients who developed metastasis within five years of RP, 69 patients (92%) were classified in either the high-risk group (58 patients) or intermediate-risk group (11 patients). The AUC of five-year survival ROC for the iPAM classifier was 0.82 (95%CI: 0.77–0.86), outperforming the AUC [0.69 (95%CI: 0.61–0.75)] for the clinical classifier assembled based on six clinical variables [Supplementary methods]. With a combined clinical and iPAM classifier, the AUC was 0.80 (95%CI: 0.75–0.85).

The AUC of survival ROC for the iPAM classifier by Gleason scores and age groups is shown in Table 4. The highest AUC of 0.87 (95%CI: 0.78–0.94) was observed in young patients (age ≤ 55 years); an intermediate AUC of 0.82 (95%CI: 0.76–0.88) was observed for middle-aged patients (age 56–70 years), and the lowest AUC of 0.69 (95%CI: 0.55–0.82) was observed in old patients (age > 70 years) [Figure 3B]. For patients with Gleason scores of 7–10, the overall AUC was 0.80 (95%CI: 0.74–0.85), with the highest AUC of 0.85 (95%CI: 0.75–0.93) in the young group and the lowest AUC of 0.67 (95%CI: 0.50–0.80) in the old group. The iPAM classifier demonstrated slightly higher prediction accuracy for patients with Gleason score of 7 compared to prediction scores for all patients.

For the MSKCC data set with no information on time of follow-up (9 patients with metastasis versus 122 without metastasis), the conventional AUC calculated with a binary metastasis status was 0.86 (95%CI: 0.73–0.99) [Supplementary Figure 2].

### Estimation of abundance of immune cell types

**Figure 4.**
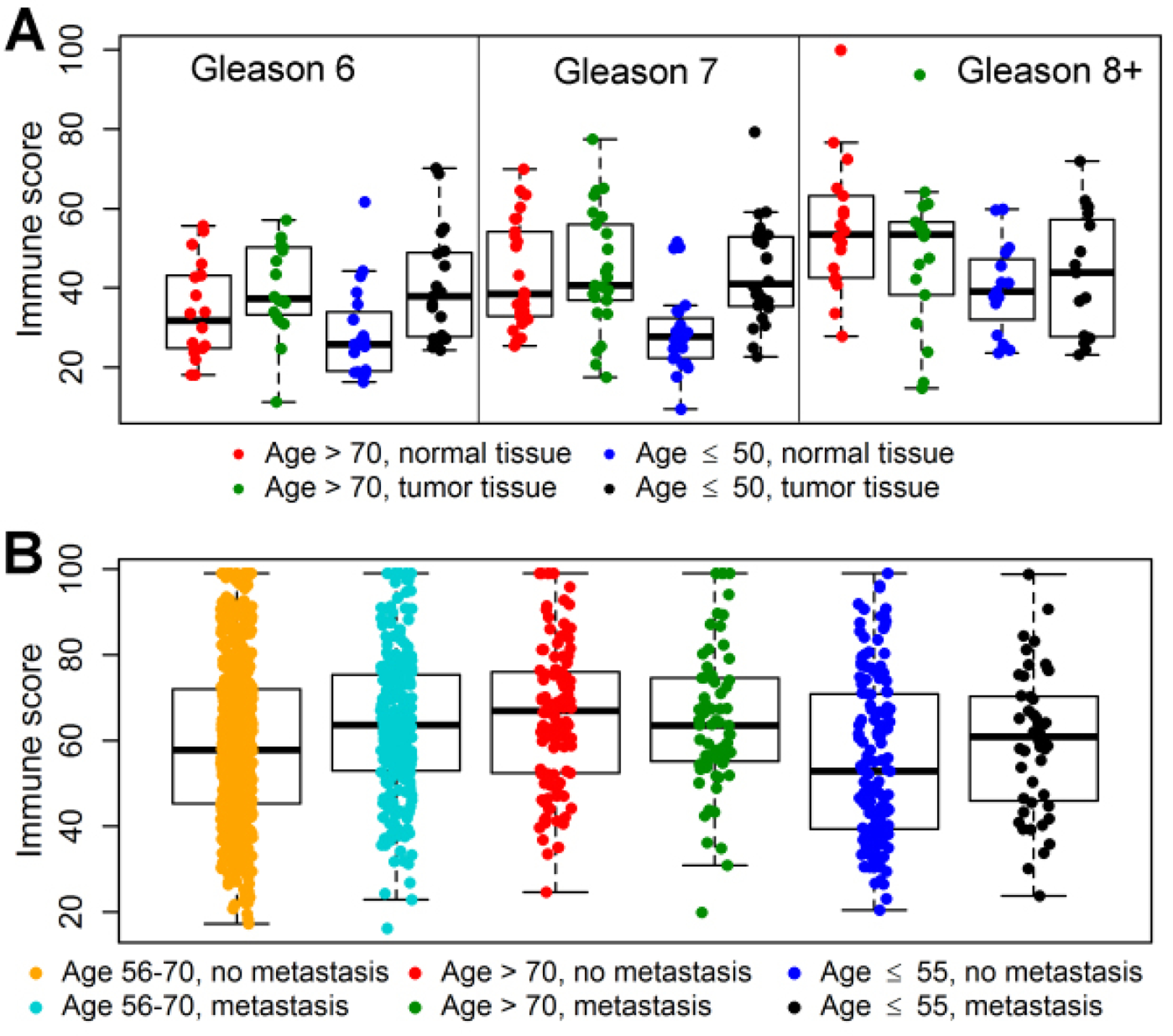
Differential abundance of immune cells (immune score) in tissue microenvironment between sample groups classified by patient age, sample type, or metastasis factors. (A) Immune scores for tumor and matched benign prostatic tissue samples from 119 patients in the COH data set were used to generate dot and box plots. Compared to matched benign prostatic tissue, tumor tissues with Gleason scores of 6 and 7 from young patients (≤ 50 years) (black) showed significantly increased abundance of immune cells (blue *vs.* black, *P* < 0.01), whereas old patients (> 70 years) showed non-significant tumor-versus-matched-benign difference in abundance of immune cells (red *vs.* green, *P* > 0.10). (B) Immune scores for 1232 primary tumor samples from five GRID data sets were used to generate plots. Compared to tumor tissues from young patients (≤ 55 years) without metastasis, tumor tissues from young patients with metastasis showed significantly (blue *vs.* black, *P* = 0.02) increased abundance of immune cell types; patients with middle age (56–70 years) also showed highly significant but a smaller with-and-without- metastasis difference (cyan *vs.* orange, *P* < 0.0001 in abundance of immune cell types than young patients (≤ 55 years); old patients (≥ 70 years) (green *vs.* red, *P* = 0.21) had no significant difference in abundance of immune cell types related to metastasis status.

The relative abundance of immune cells for tumor and matched benign prostatic tissue samples from 119 patients is displayed in Figure 4A. Compared to matched benign samples, there was a significant enrichment (*P* < 0.01, Supplementary Table 2 of immune cells in tumor samples (Gleason scores 6–7) from young patients, with no significant differences in the old patients, indicating stronger tumor-induced immune responses among young patients than among old patients. For tumors with high Gleason scores (8–10), the tumor-benign difference did not reach statistical significance.

The relative abundance of immune cells for primary tumor samples from 1,232 patients in the 5 GRID data sets is shown in Figure 4B. In young patients, there was a significantly (*P* = 0.028) greater abundance of immune cells in primary tumors from patients with metastasis compared to primary tumor samples from patients without metastasis; there were no significant metastasis-associated differences in the old patient group. In middle-aged patients, abundance of immune cells from patients with metastasis was significantly greater than that in the patients without metastasis (*P* < 0.0001, Supplementary Table 3). Four immune cell types demonstrated striking age-related differences in abundance of immune cell type [Supplementary Figure 3]. For the 687 patients from the four independent validation data sets (MC II, CC, TJU, and MSKCC), the predicted iPAM risk scores for metastasis were significantly associated with the immune scores (Spearman rho = 0.25, *P*-value = 2.68 × 10^−11^), indicating that gene expression for the 36 iPAM genes may capture information on immune responses in the tumor microenvironment that lead to metastasis.

## DISCUSSION

Prostate-specific antigen screening has reduced death from CaP due to early detection while also leading to over-treatment of low-risk CaP^[24,25]^. Three commercially available genomic classifiers (Oncotype DX^[26]^, Prolaris^[27]^, and Decipher^[15]^) are used to predict metastatic CaP and guide initial treatment and/or postoperative intervention. However, those classifiers were not designed for predicting metastatic disease in young patients and accuracy of prediction for young men with CaP was not examined. This is the first study to investigate young men with CaP as a distinct and unique patient group in both discovery and validation of prediction signatures for early metastatic disease.

The accuracy of early metastasis prediction, measured by AUC of five-year survival ROC for the Decipher in the three validation data sets (MC II, CC, and TJU) with follow-up time from date of RP are shown in Supplementary Table 4. The AUC of five-year ROC generated from the iPAM classifier was higher than that from the Decipher classifier. If only the six clinical variables were used to predict metastasis, AUC was 0.69 for both data sets; therefore, both the Decipher and iPAM classifiers showed substantial improvement on prediction of early metastasis compared to the clinical classifier alone. Inclusion of the clinical variables into the genomic classifier did not improve prediction accuracy for both classifiers. This suggests that both genomic classifiers captured the prediction information provided by the clinical variables.

To develop the iPAM classifier, we selected DEGs associated with sample-type (tumor or benign) factor, Gleason score factor, and metastasis factor. It is known that: (1) genes differentially expressed between tumor and matched benign tissues reflect the genetic basis of tumorigenesis^[28,29]^; and (2) genes differentially expressed between low and high Gleason scores correlate with tumor aggressiveness^[30]^. Therefore, in addition to being used as prognostic markers, the iPAM genes selected from those DEGs are likely to be functionally relevant to cancer progression. From Ingenuity Pathway Analysis (IPA), those DEGs were enriched in pathways of immune response, cell adhesion, and degradation of extracellular matrix [Supplementary Table 5]. Enrichment of pathways in immune response was among the up-regulated DEGs identified only in the young group (highlighted pathways in Supplementary Table 5). Estimation of abundance of immune cells in tumor and benign tissues from COH patients corroborated a role of more pronounced immune responses to tumor development in young patients than in old patients [Supplementary Table 2 and Figure 4A]. Ten of 36 iPAM genes were linked to immune-related pathways [Table 3] and showed larger metastasis-associated differences (FDR < 0.10, Supplementary Table 6 in the young group than in the old group from Decipher GRID samples [Figure 2C and Figure 2D and Supplementary Figure 4]. Furthermore, the estimated abundance of immune cells in primary tumor samples from the five Decipher GRID data sets was positively associated with the development of metastasis in young and middle-aged patients with no significant association in old patients [Supplementary Table 3 and Figure 4B]. The positive correlation between the immune scores (the abundance of immune cell types) and the risk scores of metastasis generated by the iPAM classifier for validation samples was highly significant (*P*-value = 2.68 × 10^−11^). Accuracy of early metastasis prediction from the iPAM classifier was higher for young patients (AUC = 0.87) and middle-aged patients (AUC = 0.82) than for old patients (AUC = 0.69). The trend of differences in prediction accuracy among the age groups is consistent with the observation that the metastasis-associated immune responses in the tumor microenvironment in young and middle-age patients were more pronounced than in old patients. Taken together, these findings show that more pronounced metastasis-associated immune responses in tumor microenvironment are found in young and middle-age patients than in old patients, potentially explaining the difference in accuracy of metastasis prediction among the three age groups.

This study has limitations. Although the sample sets included 58 young patients (≤ 50 years) for identifying DEGs and 197 young patients (≤ 55 years) for iPAM classifier development and validation, the sample size of young patients is modest. Second, we identified genes that serve as prognostic biomarkers and also show functional relevance to cancer progression based on IPA analysis; however, performing functional studies on the role of the 36 iPAM genes on cancer progression was beyond the scope of this study. Moreover, as previously reported, the Decipher GRID data sets may over-represent patients with adverse clinicopathologic features^[31]^.

We identified an iPAM classifier for prediction of early metastasis; the prediction accuracy of the iPAM classifier was higher for young (≤ 55 years) and middle-aged patients (56–70 years) than for old patients (> 70 years). We also provided evidence that this age-related difference in prediction accuracy can be explained by differential immune responses to metastasis development among the three age groups.

## Supplementary Material

Supplementary Material

## Figures and Tables

**Table 1.** Clinical and demographic characteristics of 119 City of Hope patients

	Total	Old (71–75 years)	Young (38–50 years)
Total patients	119	61	58
Metastatic patients	11	4	7
Mean follow-up (months)		65.3	65.8
Pathology stage			
2a	12 (0.10)	5 (0.08)	7 (0.12)
2b	2 (0.02)	0 (0)	2 (0.03)
2c	77 (0.65)	41 (0.67)	36 (0.62)
3a	19 (0.16)	10 (0.16)	9 (0.16)
3b	9 (0.07)	5 (0.08)	4 (0.07)
Gleason score			
6	37 (0.31)	18 (0.30)	19 (0.33)
7*	49 (0.41)	25 (0.41)	24 (0.41)
8 or 9	33 (0.28)	18 (0.29)	15 (0.26)
PrePSA^^^ (ng/mL)			
≤ 10.0	100 (0.84)	53 (0.87)	47 (0.81)
> 10.0	19 (0.16)	8 (0.13)	11 (0.19)
Race			
Caucasian	110 (0.92)	57 (0.93)	53 (0.91)
Asian	2 (0.02)	2 (0.03)	0
African American	6 (0.05)	2 (0.03)	4 (0.07)
Native American	1 (0.01)	0	1 (0.02)

* data for Gleason 7 patients were reported previously;

^ PSA level before surgery; PSA: Prostate specific antigen.

**Table 2.** The number of DEGs in relation to age, sample type, and Gleason score

*DEGs (|fold| > 1.5 and FDR < 0.05)	Old patients (aged 71–75)	Young patients (aged 38–50)
Tumor versus benign tissue comparison		
Patients with Gleason sum of 6	1250	1314
Patients with Gleason sum of 7	1443	1485
Patients with Gleason sum of 8+	3221	1923
^^^Low versus high Gleason score comparison	1392	650

* A total of 5156 unique DEGs identified from 8 different comparisons;

^ Low Gleason score of 6 and high Gleason score of 8+ (8 to 10). DEGs: Differentially expressed genes.

**Table 3.** Function and pathway annotation of 36 genes in the iPAM classifier

Gene name*	Bone-disease related	Immune pathway related	Cell adhesion/cell-matrix related	Cell cycle	Gene function, disease association, and pathway role (abstracted from the gene card website, https://www.genecards.org/ )
*ANO7*			Yes		Associated with advanced CaP, a target in CaP diagnosis and immunotherapy
*ANTXR1*		Yes	Yes		TLR Pathway, ECM proteins, actin cytoskeleton and promotes cell spreading
*ASPN*	Yes		Yes		Degradation of extracellular matrix (ECM) proteoglycans, associated with Osteoarthritis
*ATP5EP2*					Purine nucleotides de novo biosynthesis
*AZGP1*		Yes			Member of macroglobulin family; antigen binding; validated predictor of metastatic CaP
*C7*		Yes			Pathway of the innate immune system; involved in host immunity
*CCDC6*					Structural constituent of cytoskeleton, associated with thyroid papillary carcinoma
*CD24*		Yes			Modulates growth and differentiation signals to granulocytes and B cells
*CDC42EP5*			Yes		Induces actin filament assembly leading to cell shape changes.
*CYBA*		Yes			TNFR1 Pathway and Class I MHC mediated antigen processing and presentation
*DDIT4*		Yes			DDIT4/mTOR axis involved in the differentiation of Th17 cells
*DPT*			Yes		Cell-matrix interactions and matrix assembly, mediate adhesion
*FAM13C1*					GTPase activator activity
*FBXL8*		Yes			Involved in Class I MHC mediated antigen processing and presentation
*GLO1*	Yes				Risk factor for CaP progression and involved in bone formation and resorption
*GLYATL1*					Cytochrome P450 - arranged by substrate type and Conjugation of carboxylic acids
*GMNN*				Yes	Cell cycle regulation, increased expression plays role in colon, rectal and breast cancer
*GNPTAB*	Yes				Associated with mucolipidosis II and IIIA with low bone mineral density (osteoporosis)
*ITGBL1*			Yes		Participates in cell adhesion as and cell-surface, cell-cell and cell-matrix interaction.
*KIF21A*			Yes		Involved in microtubule transport, involved in cell polarity, and migration
*KRT15*		Yes	Yes		Involved in the structural integrity of epithelial cells; regulating innate immunity
*LBH*					Contributes to Wnt-induced tumorigenesis
*LRRN1*					Regulates Differentiation of Embryonic Stem Cells
*LTF*		Yes			Anti-inflammatory activity, induce apoptosis and inhibit proliferation in cancer cells.
*MTDH*	Yes				Promotes lung metastasis and also has an effect on bone and brain metastasis
*MYBPC1*			Yes		Contributes to the stability and maintenance of sarcomeres.
*NCAPD3*				Yes	Chromosome condensation in prometaphase and Cell Cycle, Mitotic
*NR4A1*		Yes			Involved in Class I MHC mediated antigen processing and presentation
*PCA3*					Sensitive to androgen-receptor activation, a molecular signature of prostate cancer
*RNF39*					Plays a role in an early phase of synaptic plasticity
*SFRP4*	Yes				Modulators of Wnt signaling, associated with Osteomalacia and Pyle Disease
*SLC22A3*					Disposition of small organic cations and drugs and environmental toxins
*SLC37A3*					Transporter activity and transmembrane transporter activity
*STRBP*					DNA and RNA binding, plays a role in regulation of cell growth
*TOP2A*				Yes	Associated with aggressive CaP and drug resistance; Essential in mitosis and meiosis
*UGDH*			Yes		Components of the ECM involved in cell migration, and cancer growth and metastasis

* *ANO7* and *MYBPC1* are also in the Decipher classifier^[15]^; *Top2A* is also in the Prolaris classifier^[27]^; *AZGP1* and *SFRP4* are also in the Oncotype DX classifier^[26]^; IPAM: Improved prediction analysis of microarray.

**Table 4.** AUC of five-year survival ROC for validation data stratified by Gleason score and age at diagnosis

Gleason score	Age 40–78	Age ≤ 55	Age 56–70	Age > 70
6–10	(*n* = 421, 135)* 0.82 (0.77, 0.86)	(*n* = 79, 23) 0.87 (0.78, 0.94)	(*n* = 298, 93) 0.82 (0.76, 0.88)	(*n* = 44, 19) 0.69 (0.55, 0.82)
7–10	(*n* = 357, 134) 0.80 (0.74, 0.85)	(*n* = 60, 23) 0.85 (0.75, 0.93)	(*n* = 257, 92) 0.81 (0.74, 0.86)	(*n* = 40, 19) 0.67 (0.50, 0.80)
7	(*n* = 252, 58) 0.83 (0.74, 0.90)	(*n* = 48, 12) 0.87 (NA^^^)	(*n* = 184, 41) 0.83 (0.73, 0.91)	(*n* = 20, 5) 0.79 (NA^^^)

* The number of patients without and with metastasis, respectively;

^ insufficient sample of metastatic patients to calculate 95% confidence interval by the bootstrap method.
