# Supplementary material for: Prostate cancer in young men represents a distinct clinical phenotype: gene expression signature to predict early metastases
Source: J Transl Genet Genom. Author manuscript; Available in PMC 2021 Apr 28. (PMC8081383; doi:10.20517/jtgg.2021.01)
Supplement: Supplementary Material [file NIHMS1685160-supplement-Supplementary_Material.pdf]

Supplementary methods

City of Hope patient characteristics, tissue samples, and mRNA profiling

This study was approved by the City of Hope (COH) Institutional Review Board (IRB07244). Patients with PC that were treated with RP between 1998 and 2013 at COH National Medical Center were selected based on age at diagnosis and tissue availability; in addition to 49 patients with Gleason score of 7 (3+4) reported previously[1], a total of 37 patients with Gleason score of 6 and 33 patients with Gleason score of 8–10 (Gleason 8+) were included in this study. Patient characteristics are shown in **Table 1**; a total of 61 men diagnosed between ages 71–75 years (old) and 58 men diagnosed between ages 38–50 years (young) were used to identify age-related differentially-expressed genes (DEGs) for developing a gene expression classifier to predict metastasis following RP. Tissue processing and mRNA profiling were performed as previously described[1]. Briefly, a genitourinary pathologist reviewed hematoxylin and eosin (H & E) slides and circled representative tumor or benign tissue on H&E slides from prostatectomy. Punch cores from the circled areas were obtained from the formalin-fixed paraffin-embedded blocks. RNA was extracted using RecoverAll™ Total Nucleic Acid Isolation kit (Life Technology Inc.). RNA quality was assessed using DV200 metrics, the percentage of RNA fragment size greater than 200 nucleotides, generated by running Nano chips from Agilent. RNA samples with DV200 value > 0.3 were selected for mRNA expression analysis. mRNA expression profiling of 29,000 genes (including some miRNA and LincRNA) in the human genome was generated using the Illumina Human Whole-Genome DASL (cDNA-mediated annealing, selection, extension, and ligation) HT Assay at the Genomics Core at the Case Western Reserve University. Tissue samples from patients (n = 70) with low (6) and high (8–10) Gleason score were processed and run at the same time on the cDNA-mediated Annealing, Selection, extension and Ligation (DASL) arrays (Illumina, Inc). Gene expression data for tissue samples from patients (n = 49) with Gleason score of 7 reported previously were also generated using the same Illumina DASL chips[1]. Follow-up data for the 119 COH patients were abstracted from medical records and the COH cancer registry.

Identification of age-related DEGs

DEGs [absolute fold change > 1.5 and false discovery rate (FDR) < 0.05] were identified from the DASL expression data using a mixed linear model implemented in the limma R Package[2]. In the model, sample type with two levels (tumor and benign), age group with two levels (young and old), and Gleason score with two levels [low (Gleason score 6) versus high (Gleason score of 8–10)] were categorical variables with fixed effects, and patient ID was treated as a random effect. From this model, DEGs were extracted from six comparisons: 1) Gleason-score-6 tumor versus matched benign prostatic tissue in young patient group; 2) Gleason-score-6 tumor versus matched benign prostatic tissue in old patient group; 3) Gleason-score-8 tumor versus matched benign prostatic tissue in young patient group; 4) Gleason-score-8 tumor versus matched benign prostatic tissue in old patient group; 5) Gleason-score-6 tumor versus Gleason-score-8 tumor in young patient group; and 6) Gleason-score-6 tumor versus Gleason-score-8 tumor in old patient group. the Benjamin-Hochberg (BH) method[3] was used to correct for multiple testing. In a previous study[1], we identified genes expressed differentially between Gleason-score-7 tumor and matched benign tissue in young and old patient groups. DEGs identified from these eight comparisons were considered as primary candidate genes for developing a genomic classifier to predict metastasis following radical prostatectomy (RP) [**Supplementary Figure 1**]. Ingenuity pathway analysis (IPA) of DEGs was used to predict directional biological effects (pathway involvement, cellular function, and disease association) of DEGs.

iPAM classifier development and validation

Gene expression data from primary tumors from RP in five data sets from the Decipher Genomic Resource Information Database (GRID, Decipher Biosciences, San Diego, CA) were used to develop and validate a new genomic classifier. Clinicopathological characteristics of the 1232 patients in the five datasets are shown in **Supplementary Table 1**; none of the patients had regional or distant metastatic disease at the time of RP.

Gene expression data were generated using the Affymetrix Human Exon 1.0 ST Gene Chips array and normalized as one combined data frame (46,050 genes in rows and 1232 patients in columns). A diagram of our study design is shown in **Supplementary Figure 1**.

The Mayo Clinic discovery cohort (MC I)[4] of 545 patients was used to develop the genomic classifier. In this data set, 212 patients developed early regional or distant metastasis (confirmed by bone or CT scan) within five years of biochemical relapse (BCR). This cohort was previously used to develop the Decipher classifier based on the random forest method[4]. Gene expression data from the MC I cohort for the age-related DEGs identified from the COH samples were extracted. To further reduce the DEG list, a two-sample t-test was used to only include genes differentially expressed between patients with (n = 212) and without (n = 333) metastasis. After determining the list, the 545 patients from the MC I cohort were randomly assigned into a training data set (140 with and 222 without metastasis) and a test dataset (72 with metastasis and 111 without metastasis). In order to select the optimal number of age-related DEGs that were most informative in predicting the development of metastatic prostate cancer (CaP), an improved Prediction Analysis of Microarray (iPAM) method[5-7] was applied to the training data to remove DEGs irrelevant to metastasis prediction based on minimizing the 10-fold cross-validated error rate. Specifically, the Adaptive Hierarchically Penalized Nearest Shrunken Centroid algorithm[6] was used to enable different amount of shrinkage for each variable (gene) in the process of variable selection. These iPAM-selected DEGs were assembled into an iPAM classifier by fitting a logistic regression model on the 362 samples in the training set. The iPAM classifier was then used to predict metastasis for the four independent validation data sets from the Decipher Biosciences [**Supplementary Table 1]**. The training data also were used to develop a clinical classifier including six clinical variables [pathological Gleason score (GS), preoperative PSA, seminal vesicle invasion (SVI), extra-capsular extension (ECE), lymph node invasion (LNI), and surgical margin (SM)] [**Supplementary Table 1**] as predictors in a logistic regression model to predict metastasis. An integrated classifier was also constructed

by combining informative DEGs selected by the iPAM method and the six clinical variables as predictors in a logistic regression model.

Estimation of cell-type proportion in tissue microenvironment

xCell[8] was used to estimate the enrichment score of individual cell types (21 lymphoid, 13 myeloid, 14 stromal, 9 stem, and 7 epithelial cell types) for each tissue sample using genome-wide gene expression data (> 9000 specific genes). xCell also generated an immune score representing the overall abundance of immune cells for each sample by summing the enrichment scores from all immune cell types. The DASL gene expression data from 238 COH tissue samples (119 tumor-normal pairs) and Affymetrix gene expression data from 1232 primary tumor samples from the decipher GRID were analyzed separately by the xCell method. Cell-type proportion in tissue microenvironment estimated by xCell method is a rank-based enrichment score. Non-parametric analysis of variance (ANOVA) (confidence interval and p-values generated by percentile bootstrap), implemented in the "Rallfun-v35" R codes from Dr. Wilcox[9], was used to test median differences in immune scores between sample groups classified by factors of sample type (tumor, normal), metastasis status (yes, no), and age group (old, young, middle-age).

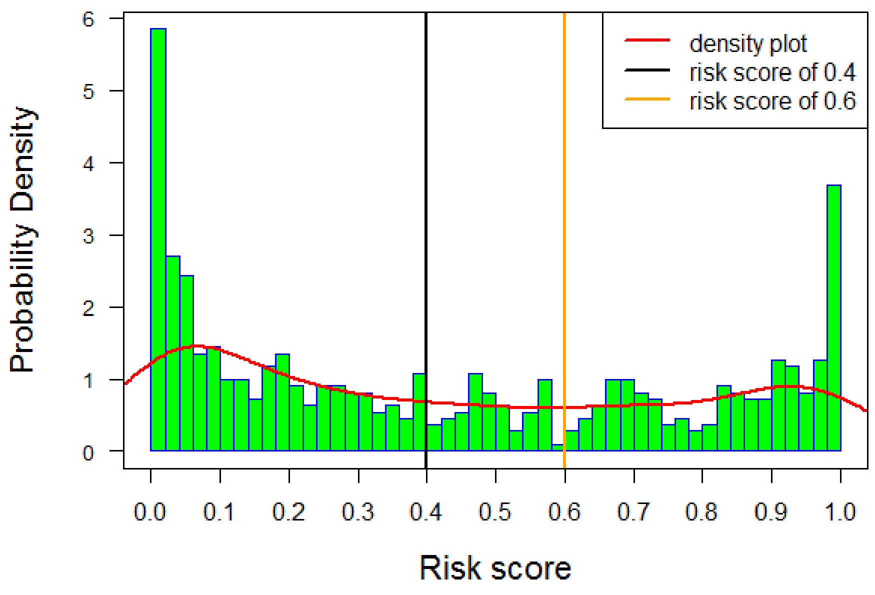

Probability Density
0
1
2
3
4
5
6
0.0
0.1
0.2
0.3
0.4
0.5
0.6
0.7
0.8
0.9
1.0
Risk score
density plot
risk score of 0.4
risk score of 0.6

Supplementary Figure 1. Bimodal distribution of predicted risk scores for developing metastasis generated by the iPAM classifier

Risk scores for 556 samples from the three Decipher GRID validation data sets (MC II, CC, and TJU) with follow-up time were used to generate this histogram. The predicted iPAM risk scores for metastasis showed a bimodal distribution with score range of 0–1 where higher scores represent higher risk of developing metastasis. Based on the distribution, risk scores of 0.4 and 0.6 were selected to stratify risk scores into three groups, low risk group [0–0.4), intermediate risk group [0.4–0.6], and high risk group (0.6–1].

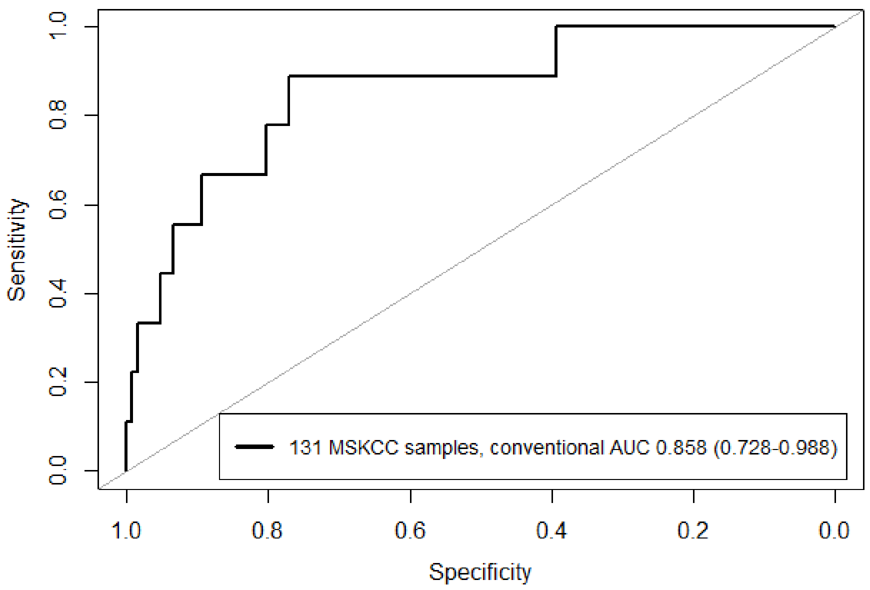

Supplementary Figure 2. High conventional area under the curve (AUC) of receiver operating characteristic (ROC) for 131 samples from the Memorial Sloan Kettering Cancer Center (MSKCC) validation data set.

For the MSKCC data set (the fourth validation data, 131 patients in total and 9 patients with metastasis) with no information on follow-up time, the conventional AUC for iPAM (improved Prediction Analysis of Microarray) classifier calculated based on binary metastasis status was 0.86 with 95% CI of 0.73–0.99.

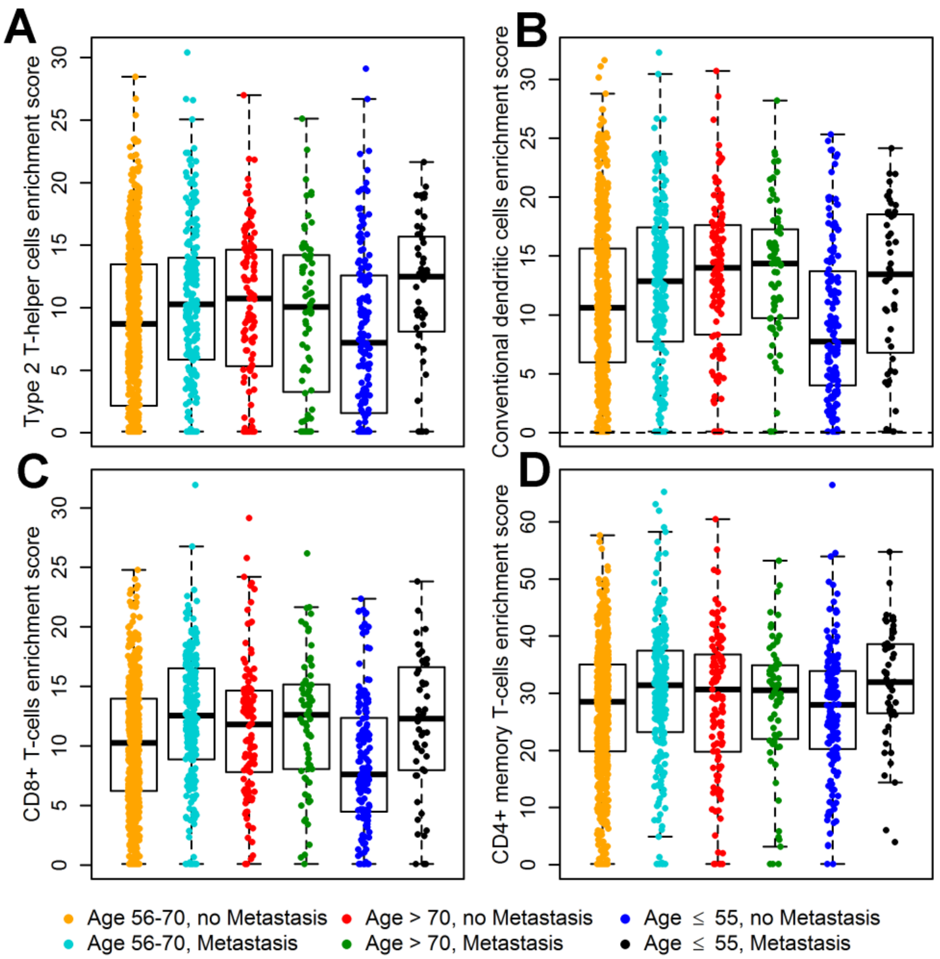

Supplementary Figure 3. Metastasis-associated differential abundance (enrichment scores) of four specific immune cell types among three age groups.

Plots were generated using the cell type enrichment scores estimated for the 1232 primary tumor samples from the five Decipher GRID data sets. Out of 33 immune cell types, 4 specific cell types, Type 2 T helper cells (A), Conventional dendritic cells (B), CD8+ T cells (C), and CD4+ memory T cells (D), in young and middle age group (≤ 55 yeas) demonstrated significantly ($P < 0.05$) greater abundance of immune cells in primary tumors from patients with metastasis compared to primary tumor samples from patients without metastasis; but there were no significant differences in the old patient group (>70 years). (Statistical p-values and median differences in abundance of immune cell type between metastasis and without metastasis groups among three age groups are included in the following table).

Table specific to foot note description in Supplementary Figure 3: Statistical testing of mean difference in abundance of immune cell types between patients with and without metastasis.

Figure Panel	Immune cell type	Young(≤ 55 years, n = 197)		Middle (56–70 years, n = 846)		Old (> 70 years, n = 189)	
		median difference(CI)	p-value	median difference (CI)	p-value	median difference (CI)	p-value
A	Type 2 T-helper cells	4.7(2.0–7.2)	< 0.001	1.7 (0.5–3.0)	0.003	-0.6 (-3.4–2.3)	0.701
B	Conventional dendritic cells	5.5(2.1–8.6)	0.002	2.3(1.1–3.6)	0.001	0.16 (-2.2–2.0)	0.302
C	CD8+ T-cells	4.5(2.1–6.9)	< 0.001	2.3(1.3–3.4)	< 0.001	1.0 (-1.1–3.1)	0.322
D	CD4+ memory T-cells	4.1(0.5–8.3)	0.027	2.6(0.9–4.2)	0.005	0.01 (-3.8–4.4)	0.981

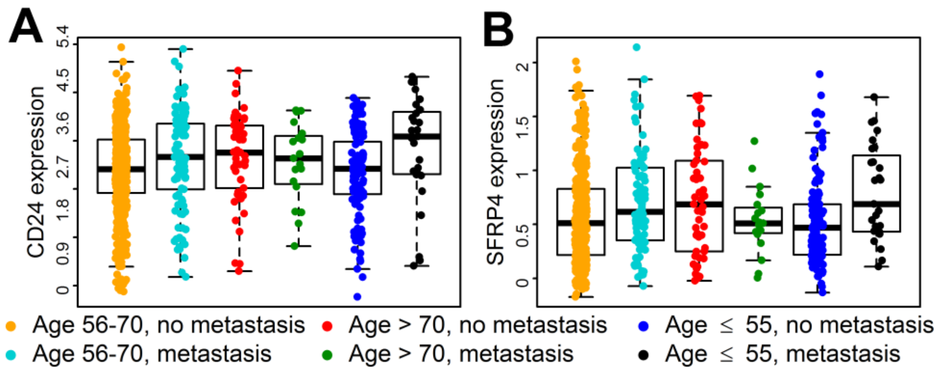

Supplementary Figure 4. Boxplots and dotplots of age-related DEGs between patients with and without metastasis.

CD24 (CD24 antigen) and *SFRP4* (Secreted Frizzled Related Protein 4) are among the 36 genes for iPAM classifier. Gene expression data for 687 patients from four validation data sets (MC II, CC, TJU, MSK) were used to generate plots. Among young patients (≤ 55 years), significantly increased median expression levels for CD24 gene (A) and SFRP4 (B) were observed in patients with metastasis (black) compared to patients without metastasis (blue) (Benjamini-Hochberg adjusted $p < 0.05$); however, there was no significant median expression difference related to metastasis status among patients older than 70 years (red and green) ($p > 0.16$). The gene expression difference related to metastasis status was significant among patients with age between 56 and 70 years (Benjamini-Hochberg adjusted $p < 0.05$), but a smaller median difference was observed compared to young patients (≤ 55).

Supplementary Table 1. Patient characteristics for discovery and validation data obtained from the Decipher GRID data sets

	Total (n)	Discovery data: MC I*			Total (n)	Validation data sets: MC II, CC, TJU, MSKCC*		
		Age > 70	Age 56-70	Age ≤ 55		Age > 70	Age 56-70	Age ≤ 55
**Total cases**	545	121 (22)	372 (68)	52 (10)	687	68 (10)	474 (69)	145 (21)
Number with metastasis^	212	49 (23)	142 (67)	21 (10)	144	19 (13)	98 (68)	27 (19)
Mean follow-up (month)		172	189	186		79	95	86
**Pre-operative PSA**								
<10	282	57 (20)	193 (69)	2 (11)	344	44 (13)	241 (70)	59 (17)
≥ 20	248	61 (24)	168 (68)	9 (8)	202	19 (9)	141 (70)	42 (21)
**Gleason score**								
low (5 or 6)	63	10 (16)	47 (74)	(10)	105	6 (6)	66 (63)	33 (31)
intermediate (7)	271	62 (23)	177 (65)	32 (12)	384	27 (7)	273 (71)	84 (22)
high (8–10)	211	49 (23)	148 (70)	4 (7)	196	35 (18)	134 (68)	27 (14)
**Pathologic T**								
pT2a	80	17 (21)	56 (70)	(9)	33	6 (18)	18 (55)	9 (27)
pT2b	139	33 (24)	92 (66)	14 (10)	123	13 (11)	83 (67)	27 (22)
pT2c	0	0	0	0	36	0	29 (81)	7 (19)
pT3a	132	23 (17)	100 (76)	9 (7)	134	10 (8)	90 (67)	34 (25)
pT3b	121	39 (32)	72 (60)	10 (8)	130	16 (12)	89 (69)	25 (19)
pT3c	0	0	0	0	2	1 (50)	0	1 (50)
pT4	0	0	0	0	14	1 (7)	7 (50)	6 (43)
pTx	73	9 (12)	52 (72)	12 (16)	33	6 (18)	17 (52)	10 (30)
**Seminal vesicle invasion**	176	46 (26)	112 (64)	18 (10)	182	24 (13)	120 (66)	38 (21)
**Extra-capsular extension**	273	60 (22)	190 (70)	23 (8)	389	35 (9)	272 (70)	82 (21)
**Lymph node invasion**	73	9 (12)	52 (72)	12 (16)	41	6 (15)	23 (56)	12 (29)
**Surgical margins**	266	60 (23)	179 (67)	27 (10)	361	31 (9)	248 (69)	82 (23)
**Androgen deprivation therapy**	320	76 (24)	217 (68)	27 (8)	166	24 (14)	110 (67)	31 (19)
**Radiation therapy**	148	31 (21)	102 (69)	15 (10)	255	23 (9)	168 (66)	63 (25)

*MC I = Mayo Clinic data set I; MC II = Mayo Clinic data set II; CC = Cleveland Clinic data set; TJU = Thomas Jefferson University data set; MSKCC = Memorial Sloan-Kettering Cancer Center data set, PSA= Prostate-Specific Antigen; **Row percent in parentheses

^Metastasis is referred to as regional or distant metastasis for the Mayo Clinic data set I (MC I) and distant metastasis for the four validation data set (MC II, CC, TJU, MSKCC)

Supplementary Table 2. Median difference in immune score between tumor and matched benign prostatic samples from the 119 COH patients

Gleason score	Age group	Number of patients	Median in tissue group		Median difference	95% CI of median difference	p-value*
			tumor	benign			
6	age > 70	18	39.53	31.57	7.96	-2.94–17.42	0.150
	age ≤ 50	19	37.95	26.10	11.85	3.99–20.39	0.004
7	age > 70	25	42.02	40.05	1.97	-7.72–11.51	0.692
	age ≤ 45	24	42.67	27.46	15.21	8.01–23.55	< 0.001
8	age > 70	18	51.38	53.48	-2.10	-13.49–5.18	0.602
	age ≤ 50	15	42.55	39.54	3.01	-6.62–14.27	0.596

*The Dqcomhd function for paired samples in the WRS (Rallfun-v35) package[9] was used to test significance of median difference in immune score between tumors and matched benign prostatic samples. For paired samples, it uses the quantile estimator and bootstrapping to determine the confidence interval (CI) for median difference and the p-values (corrected for multiple testing).

Supplementary Table 3 Median difference in immune score between patients with and without metastasis in three age groups from GRID data sets

Age (years) group	N*	Median in patient group		Median difference	95% CI of median difference	p-value**
		with metastasis	without metastasis			
age > 70	189	62.75	66.32	-3.56	-8.44–1.89	0.204
age 56-70	846	63.51	57.88	5.63	3.06–8.22	< 0.001
age ≤ 55	197	60.93	52.22	8.71	0.86–14.66	0.028

*N: number of patients in each age group from five GRID data sets

**The pb2gen function in the WRS (Rallfun-v35) package[9] was used to test significance of median difference in immune score between patients with and without metastasis.

Supplementary Table 4. AUC of ROC for Decipher risk scores for metastasis-free survival within five years of RP in the validation group stratified by Gleason score and age factors.

Gleason score	Age 40 - 78	Age ≤ 55	Age 56 - 70	Age ≤ 70	Age > 70
6 to 10	(n* = 75, 60, 421)<br>0.80 (0.75, 0.86)	(n = 16, 7, 79)<br>0.90 (0.79, 0.98)	(n = 46, 47, 298)<br>0.80 (0.73, 0.86)	(n = 62, 54, 377)<br>0.82 (0.77, 0.88)	(n = 13, 6, 44)<br>0.69 (0.52, 0.85)
7 to 10	(n = 75, 59, 357)<br>0.79 (0.74, 0.84)	(n = 16, 7, 60)<br>0.89 (0.78, 0.97)	(n = 46, 46, 257)<br>0.78 (0.71, 0.85)	(n = 62, 53, 317)<br>0.81 (0.75, 0.81)	(n = 13, 6, 40)<br>0.68 (0.48, 0.83)
7	(n = 32, 26, 252)<br>0.79 (0.71, 0.86)	(n = 6, 6, 48)<br>0.90 (NA^)	(n = 22, 19, 184)<br>0.75 (0.64, 0.84)	(n = 28, 25, 232)<br>0.78 (0.70, 0.86)	(n = 4, 1, 20)<br>0.80 (NA^)

*n: number of patients with early metastasis in five year of RP, late metastasis after five year of RP , and without metastasis at last follow up, respectively; ^ NA, due to few metastatic patients, not able to calculate 95% confidence interval by a bootstrap method

Supplementary Table 5. Ingenuity pathway analysis (IPA) of differentially expressed genes (DEGs).

Top-five IPA results for the 179 up-regulated DEGs (Fold change > 2.0 and FDR < 0.05) identified from Gleason-6 tumor versus matched benign prostatic tissue comparison in old patient group

Top Canonical Pathways	p-value	Overlap
Epithelial Adherens Junction Signaling	2.34E-04	4.6% 7/153
Atherosclerosis Signaling	4.89E-04	4.8% 6/124
FXR/RXR Activation	5.32E-04	4.8% 6/126
Hepatic Fibrosis/Hepatic Stellate Cell Activation	7.58E-04	3.8% 7/186
Top Diseases and Disorders	p-value range	Number of Genes
Cancer	4.94E-04 - 1.90E-13	175
Organismal Injury and Abnormalities	4.94E-04 - 1.90E-13	175
Gastrointestinal Disease	4.82E-04 - 1.04E-10	160
Dermatological Diseases and Conditions	3.74E-04 - 2.43E-10	122
Reproductive System Disease	4.94E-04 - 6.91E-08	115
Molecular and Cellular Functions	p-value range	Number of Genes
Cellular Assembly and Organization	4.29E-04 - 1.42E-05	42
Cell Cycle	1.89E-04 - 3.69E-05	7
Cellular Movement	3.77E-04 - 4.09E-05	13
Cellular Development	4.14E-04 - 7.54E-05	48
Cellular Growth and Proliferation	3.14E-04 - 7.54E-05	43
Physiological System Development and Function	p-value range	Number of Genes
Hair and Skin Development and Function	9.48E-06 - 9.48E-06	5
Skeletal and Muscular Development and Function	4.03E-04 - 1.15E-05	29
Organismal Development	4.90E-04 - 1.29E-05	54
Connective Tissue Development and Function	1.89E-04 - 3.35E-05	16
Tissue Development	4.03E-04 - 3.35E-05	33

Top-five IPA results for the 320 down-regulated DEGs (Fold change > 2.0 and FDR < 0.05) identified from Gleason-6 tumor versus matched benign prostatic tissue comparison in old patient group

Top Canonical Pathways	p-value	Overlap
Nicotine Degradation II	3.62E-05	10.8% 7/65
Nicotine Degradation III	1.36E-04	10.7% 6/56
Estrogen Biosynthesis	2.69E-04	12.2% 5/41
Bupropion Degradation	3.91E-04	16.0% 4/25
Human Embryonic Stem Cell Pluripotency	7.14E-04	5.9% 8/136

Top Diseases and Disorders	p-value range	Number of Genes
Cancer	2.20E-03 - 3.40E-14	297
Organismal Injury and Abnormalities	2.20E-03 - 3.40E-14	300
Dermatological Diseases and Conditions	1.73E-03 - 5.79E-12	220
Gastrointestinal Disease	1.87E-03 - 4.13E-10	271
Endocrine System Disorders	1.71E-03 - 3.86E-09	259

Molecular and Cellular Functions	p-value range	Number of Genes
Cellular Development	2.06E-03 - 2.14E-08	125
Cellular Movement	2.11E-03 - 1.81E-07	99
Cell Morphology	1.93E-03 - 6.16E-07	79
Cellular Assembly and Organization	1.93E-03 - 8.51E-06	72
Cellular Function and Maintenance	1.85E-03 - 8.51E-06	75

Physiological System Development and Function	p-value range	Number of Genes
Embryonic Development	2.05E-03 - 6.13E-14	88
Hair and Skin Development and Function	2.02E-03 - 6.13E-14	54
Organ Development	2.02E-03 - 6.13E-14	76
Organismal Development	2.12E-03 - 6.13E-14	124
Tissue Development	2.02E-03 - 6.13E-14	122

Top-five IPA results for the 192 up-regulated DEGs (Fold change > 2.0 and FDR < 0.05) identified from Gleason-6 tumor versus matched benign prostatic tissue in young patient group

Top Canonical Pathways	p-value	Overlap
Atherosclerosis Signaling	9.54E-05	5.6% 7/124
Granulocyte Adhesion and Diapedesis	1.56E-04	4.5% 8/179
Bladder Cancer Signaling	1.85E-04	6.2% 6/97
Agranulocyte Adhesion and Diapedesis	2.51E-04	4.2% 8/192
Inhibition of Matrix Metalloproteases	3.34E-04	10.3% 4/39

Top Diseases and Disorders	p-value range	Number of Genes
Dermatological Diseases and Conditions	1.48E-03 - 2.55E-11	129
Organismal Injury and Abnormalities	1.80E-03 - 2.55E-11	187
Cancer	1.76E-03 - 3.32E-11	185
Gastrointestinal Disease	1.80E-03 - 3.21E-09	169
Immunological Disease	1.71E-03 - 1.31E-07	51

Molecular and Cellular Functions	p-value range	Number of Genes
Cellular Movement	2.41E-04 - 1.01E-08	51
Cell Death and Survival	1.00E-04 - 1.09E-07	54

Carbohydrate Metabolism	2.38E-04 - 6.63E-06	16
Small Molecule Biochemistry	2.00E-04 - 6.63E-06	8
Cellular Assembly and Organization	1.00E-04 - 7.14E-06	29

Physiological System Development and Function	p-value range	Number of Genes
Cell-mediated Immune Response	1.52E-03 - 2.50E-07	11
Hematological Development and Function	1.71E-03 - 2.50E-07	38
Immune Cell Trafficking	1.71E-03 - 2.50E-07	33
Tissue Morphology	1.48E-03 - 1.68E-06	59
Organismal Development	1.54E-03 - 5.51E-06	56

Top-five IPA results for the 322 down-regulated DEGs (Fold change > 2.0 and FDR < 0.05) identified from Gleason-6 tumor versus matched benign prostatic tissue in young patient group

Top Canonical Pathways	p-value	Overlap
Nicotine Degradation III	1.40E-04	10.7% 6/56
Estrogen Biosynthesis	2.77E-04	12.2% 5/41
Nicotine Degradation II	3.21E-04	9.2% 6/65
Bupropion Degradation	4.00E-04	16.0% 4/25
Amyotrophic Lateral Sclerosis Signaling	5.03E-04	7.1% 7/98

Top Diseases and Disorders	p-value range	Number of Genes
Cancer	4.32E-03 - 1.08E-13	302
Organismal Injury and Abnormalities	4.32E-03 - 1.08E-13	304
Dermatological Diseases and Conditions	3.77E-03 - 2.61E-10	219
Gastrointestinal Disease	3.85E-03 - 7.57E-10	273
Endocrine System Disorders	3.84E-03 - 1.04E-07	258

Molecular and Cellular Functions	p-value range	Number of Genes
Cellular Assembly and Organization	4.03E-03 - 1.98E-07	68
Cell-To-Cell Signaling and Interaction	2.91E-03 - 3.32E-07	49
Cellular Development	4.03E-03 - 9.00E-07	104
Lipid Metabolism	4.03E-03 - 5.09E-06	47
Molecular Transport	4.03E-03 - 5.09E-06	76

Physiological System Development and Function	p-value range	Number of Genes
Embryonic Development	4.04E-03 - 1.18E-08	88
Hair and Skin Development and Function	2.93E-03 - 1.18E-08	31
Organ Development	2.40E-03 - 1.18E-08	69
Organismal Development	4.08E-03 - 1.18E-08	128
Tissue Development	4.26E-03 - 1.18E-08	113

Top-five IPA results for the 107 up-regulated DEGs (Fold change > 2.0 and FDR < 0.05) identified from Gleason-7 tumor versus matched benign prostatic tissue in old patient group

Top Canonical Pathways	p-value	Overlap
SPINK1 Pancreatic Cancer Pathway	1.89E-04	6.7% 4/60
GDP-L-fucose Biosynthesis I (from GDP-Dmannose)	9.45E-03	50.0% 1/2
Intrinsic Prothrombin Activation Pathway	1.69E-02	4.8% 2/42
Neuroprotective Role of THOP1 in Alzheimer's Disease	1.78E-02	2.6% 3/116
LPS/IL-1 Mediated Inhibition of RXR Function	2.20E-02	1.8% 4/224
Top Diseases and Disorders	p-value range	Number of Genes
Cancer	3.61E-03 - 5.78E-10	104
Organismal Injury and Abnormalities	3.61E-03 - 5.78E-10	104
Gastrointestinal Disease	3.49E-03 - 5.97E-09	98
Reproductive System Disease	3.61E-03 - 3.34E-06	68
Renal and Urological Disease	3.61E-03 - 7.60E-05	11
Molecular and Cellular Functions	p-value range	Number of Genes
Cellular Movement	8.89E-04 - 8.89E-04	5
Cellular Development	1.42E-03 - 1.42E-03	8
Cellular Growth and Proliferation	1.42E-03 - 1.42E-03	8
Carbohydrate Metabolism	2.81E-03 - 1.95E-03	6
Nucleic Acid Metabolism	1.95E-03 - 1.95E-03	2
Physiological System Development and Function	p-value range	Number of Genes
Organ Development	3.50E-03 - 7.83E-04	8
Tissue Morphology	3.24E-03 - 2.81E-03	5
Embryonic Development	3.50E-03 - 3.24E-03	7
Organismal Development	3.50E-03 - 3.24E-03	7
Nervous System Development and Function	3.61E-03 - 3.50E-03	7

Top-five IPA results for the 195 down-regulated DEGs (Fold change > 2.0 and FDR < 0.05) identified from Gleason-7 tumor versus matched benign prostatic tissue in old patient group

Top Canonical Pathways	p-value	Overlap
Hepatic Fibrosis / Hepatic Stellate Cell Activation	4.16E-05	4.8% 9/186
PCP pathway	2.00E-04	8.2% 5/61
PXR/RXR Activation	2.70E-04	7.7% 5/65

LPS/IL-1 Mediated Inhibition of RXR Function	8.57E-04	3.6% 8/224
Glutathione Redox Reactions I	1.19E-03	12.5% 3/24

Top Diseases and Disorders	p-value range	Number of Genes
Cancer	9.63E-04 - 2.49E-11	187
Organismal Injury and Abnormalities	9.63E-04 - 2.49E-11	190
Reproductive System Disease	9.63E-04 - 5.69E-09	142
Skeletal and Muscular Disorders	9.30E-04 - 1.46E-08	78
Dermatological Diseases and Conditions	9.17E-04 - 4.67E-08	141

Molecular and Cellular Functions	p-value range	Number of Genes
Cellular Development	9.04E-04 - 4.69E-09	86
Cell Death and Survival	6.53E-04 - 8.87E-08	84
Cell Morphology	7.57E-04 - 1.27E-07	63
Cellular Assembly and Organization	9.66E-04 - 1.27E-07	56
Cellular Function and Maintenance	7.79E-04 - 1.27E-07	49

Physiological System Development and Function	p-value range	Number of Genes
Embryonic Development	9.04E-04 - 4.19E-10	83
Organismal Development	9.30E-04 - 4.19E-10	107
Hair and Skin Development and Function	3.76E-04 - 1.00E-09	29
Organ Development	8.59E-04 - 1.00E-09	73
Tissue Development	9.30E-04 - 1.00E-09	101

Top-five IPA results for the 131 up-regulated DEGs (Fold change > 2.0 and FDR < 0.05) identified from Gleason-7 tumor versus matched benign prostatic tissue in young patient group

Top Canonical Pathways	p-value	Overlap
Agranulocyte Adhesion and Diapedesis	1.71E-05	4.2% 8/192
Granulocyte Adhesion and Diapedesis	8.71E-05	3.9% 7/179
Atherosclerosis Signaling	8.82E-05	4.8% 6/124
Inhibition of Matrix Metalloproteases	1.51E-03	7.7% 3/39
Bladder Cancer Signaling	2.50E-03	4.1% 4/97

Top Diseases and Disorders	p-value range	Number of Genes
Cancer	2.42E-04 - 9.63E-11	127
Organismal Injury and Abnormalities	2.42E-04 - 9.63E-11	129
Gastrointestinal Disease	2.39E-04 - 1.20E-08	117
Immunological Disease	2.39E-04 - 3.19E-08	41
Reproductive System Disease	2.37E-04 - 1.10E-07	91

Molecular and Cellular Functions	p-value range	Number of Genes
Cellular Movement	2.41E-04 - 1.01E-08	51

Cell Death and Survival	1.00E-04 - 1.09E-07	54
Carbohydrate Metabolism	2.38E-04 - 6.63E-06	16
Small Molecule Biochemistry	2.00E-04 - 6.63E-06	8
Cellular Assembly and Organization	1.00E-04 - 7.14E-06	29

Physiological System Development and Function	p-value range	Number of Genes
Tissue Morphology	2.37E-04 - 2.62E-06	39
Hematological Development and Function	2.41E-04 - 3.16E-06	26
Immune Cell Trafficking	2.41E-04 - 3.16E-06	24
Cardiovascular Development and Function	2.12E-04 - 5.60E-06	23
Organismal Development	2.00E-04 - 5.60E-06	39

Top-five IPA results for the 212 down-regulated DEGs (Fold change > 2.0 and FDR < 0.05) identified from Gleason-7 tumor versus matched benign prostatic tissue in young patient group

Top Canonical Pathways	p-value	Overlap
Glutathione-mediated Detoxification	2.25E-04	12.5% 4/32
LPS/IL-1 Mediated Inhibition of RXR Function	2.88E-04	4.0% 9/224
PXR/RXR Activation	3.71E-04	7.7% 5/65
Histamine Degradation	5.10E-04	17.6% 3/17
Glutathione Redox Reactions I	1.45E-03	12.5% 3/24

Top Diseases and Disorders	p-value range	Number of Genes
Cancer	1.80E-03 - 9.91E-11	201
Organismal Injury and Abnormalities	1.83E-03 - 9.91E-11	204
Dermatological Diseases and Conditions	1.17E-03 - 1.99E-10	150
Gastrointestinal Disease	1.83E-03 - 6.99E-08	181
Reproductive System Disease	5.25E-04 - 2.80E-07	136

Molecular and Cellular Functions	p-value range	Number of Genes
Cellular Assembly and Organization	1.80E-03 - 4.84E-07	60
Cellular Function and Maintenance	1.30E-03 - 4.84E-07	56
Cell Morphology	1.80E-03 - 1.01E-06	59
Cell-To-Cell Signaling and Interaction	1.80E-03 - 1.36E-06	47
Cell Death and Survival	1.31E-03 - 1.38E-06	76

Physiological System Development and Function	p-value range	Number of Genes
Embryonic Development	1.81E-03 - 2.61E-09	61
Hair and Skin Development and Function	1.30E-03 - 2.61E-09	29
Organ Development	1.81E-03 - 2.61E-09	55
Organismal Development	1.84E-03 - 2.61E-09	94
Tissue Development	1.81E-03 - 2.61E-09	91

Top-five IPA results for the 384 up-regulated DEGs (Fold change > 2.0 and FDR < 0.05) identified from Gleason-8 tumor versus matched benign prostatic tissue in old patient group

Top Canonical Pathways	p-value	Overlap
Intrinsic Prothrombin Activation Pathway	7.07E-05	14.3% 6/42
D-mannose Degradation	1.69E-02	100.0% 1/1
Atherosclerosis Signaling	1.90E-02	4.8% 6/124
Fatty Acid Activation	1.97E-02	15.4% 2/13
Oleate Biosynthesis II (Animals)	1.97E-02	15.4% 2/13
Top Diseases and Disorders	p-value range	Number of Genes
Cancer	1.49E-02 - 1.67E-24	368
Organismal Injury and Abnormalities	1.56E-02 - 1.67E-24	371
Gastrointestinal Disease	1.39E-02 - 4.85E-19	335
Endocrine System Disorders	1.56E-02 - 1.12E-15	310
Dermatological Diseases and Conditions	1.36E-02 - 6.55E-10	229
Molecular and Cellular Functions	p-value range	Number of Genes
Cell Morphology	1.47E-02 - 6.40E-05	54
Cellular Assembly and Organization	1.47E-02 - 6.40E-05	68
Cellular Development	1.49E-02 - 6.40E-05	55
Cellular Function and Maintenance	1.42E-02 - 6.40E-05	64
Cellular Growth and Proliferation	1.49E-02 - 6.40E-05	47
Physiological System Development and Function	p-value range	Number of Genes
Nervous System Development and Function	1.52E-02 - 9.81E-07	93
Embryonic Development	1.52E-02 - 1.45E-06	83
Organ Development	1.49E-02 - 1.45E-06	54
Organismal Development	1.56E-02 - 1.45E-06	115
Tissue Development	1.49E-02 - 1.45E-06	85

Top-five IPA results for the 719 down-regulated DEGs (Fold change > 2.0 and FDR < 0.05) identified from Gleason-8 tumor versus matched benign prostatic tissue in old patient group

Top Canonical Pathways	p-value	Overlap
Human Embryonic Stem Cell Pluripotency	1.29E-08	14.7% 20/136
Hepatic Fibrosis / Hepatic Stellate Cell Activation	3.11E-08	12.4% 23/186
Axonal Guidance Signaling	1.59E-07	8.0% 39/487
Osteoarthritis Pathway	1.77E-05	9.4% 20/213
Clathrin-mediated Endocytosis Signaling	1.73E-04	8.8% 17/194
Top Diseases and Disorders	p-value range	Number of Genes

Cancer	3.34E-06 - 1.74E-31	683
Organismal Injury and Abnormalities	3.60E-06 - 1.74E-31	689
Dermatological Diseases and Conditions	1.82E-06 - 9.89E-29	504
Reproductive System Disease	3.34E-06 - 2.78E-26	497
Gastrointestinal Disease	3.60E-06 - 4.47E-21	617
Molecular and Cellular Functions	p-value range	Number of Genes
Cellular Movement	2.11E-06 - 9.21E-17	218
Cell-To-Cell Signaling and Interaction	1.90E-06 - 8.46E-14	144
Cellular Assembly and Organization	1.58E-06 - 8.46E-14	157
Cellular Development	3.67E-06 - 4.24E-12	259
Cellular Growth and Proliferation	2.68E-06 - 4.24E-12	245
Physiological System Development and Function	p-value range	Number of Genes
Organismal Development	3.51E-06 - 2.56E-18	338
Cardiovascular System Development and Function	3.69E-06 - 9.63E-18	179
Embryonic Development	2.34E-06 - 5.82E-15	233
Hair and Skin Development and Function	2.34E-06 - 5.82E-15	83
Organ Development	2.34E-06 - 5.82E-15	207

Top-five IPA results for the 279 up-regulated DEGs (Fold change > 2.0 and FDR < 0.05) identified from Gleason-8 tumor versus matched benign prostatic tissue in young patient group

Top Canonical Pathways	p-value	Overlap*
Cell Cycle: G2/M DNA Damage Checkpoint Regulation	3.29E-04	10.2% 5/49
Atherosclerosis Signaling	8.63E-04	5.6% 7/124
Mitotic Roles of Polo-Like Kinase	1.30E-03	7.6% 5/66
Intrinsic Prothrombin Activation Pathway	1.72E-03	9.5% 4/42
Cell Cycle Control of Chromosomal Replication	4.94E-03	7.1% 4/56
Top Diseases and Disorders	p-value range	Number of Genes
Cancer	4.30E-03 - 9.30E-19	267
Organismal Injury and Abnormalities	4.30E-03 - 9.30E-19	268
Gastrointestinal Disease	4.00E-03 - 3.22E-15	246
Endocrine System Disorders	3.86E-03 - 2.47E-10	224
Hepatic System Disease	2.70E-03 - 3.95E-10	153
Molecular and Cellular Functions	p-value range	Number of Genes
Cell Cycle	4.00E-03 - 1.52E-11	49
Cellular Assembly and Organization	4.16E-03 - 5.10E-09	77
DNA Replication, Recombination, and Repair	2.42E-03 - 5.10E-09	29
Cell-To-Cell Signaling and Interaction	4.16E-03 - 9.56E-09	44

Cell Death and Survival	3.93E-03 - 2.99E-07	99
**Physiological System Development and Function**	**p-value range**	**Number of Genes**
Nervous System Development and Function	4.00E-03 - 6.47E-08	66
Tissue Morphology	3.58E-03 - 6.47E-08	35
Digestive System Development and Function	4.00E-03 - 6.23E-05	17
Embryonic Development	4.00E-03 - 6.23E-05	64
Endocrine System Development and Function	3.58E-03 - 6.23E-05	10

Top-five IPA results for the 392 down-regulated DEGs (Fold change > 2.0 and FDR < 0.05) identified from Gleason-8 tumor versus matched benign prostatic tissue in young patient group

Top Canonical Pathways	p-value	Overlap*
Cell Cycle: G2/M DNA Damage Checkpoint Regulation	3.29E-04	10.2 % 5/49
Atherosclerosis Signaling	8.63E-04	5.6 % 7/124
Mitotic Roles of Polo-Like Kinase	1.30E-03	7.6 % 5/66
Intrinsic Prothrombin Activation Pathway	1.72E-03	9.5 % 4/42
Cell Cycle Control of Chromosomal Replication	4.94E-03	7.1 % 4/56
**Top Diseases and Disorders**	**p-value range**	**Number of Genes**
Cancer	4.30E-03 - 9.30E-19	267
Organismal Injury and Abnormalities	4.30E-03 - 9.30E-19	268
Gastrointestinal Disease	4.00E-03 - 3.22E-15	246
Endocrine System Disorders	3.86E-03 - 2.47E-10	224
Hepatic System Disease	2.70E-03 - 3.95E-10	153
**Molecular and Cellular Functions**	**p-value range**	**Number of Genes**
Cell Cycle	4.00E-03 - 1.52E-11	49
Cellular Assembly and Organization	4.16E-03 - 5.10E-09	77
DNA Replication, Recombination, and Repair	2.42E-03 - 5.10E-09	29
Cell-To-Cell Signaling and Interaction	4.16E-03 - 9.56E-09	44
Cell Death and Survival	3.93E-03 - 2.99E-07	99
**Physiological System Development and Function**	**p-value range**	**Number of Genes**
Nervous System Development and Function	4.00E-03 - 6.47E-08	66
Tissue Morphology	3.58E-03 - 6.47E-08	35
Digestive System Development and Function	4.00E-03 - 6.23E-05	17
Embryonic Development	4.00E-03 - 6.23E-05	64
Endocrine System Development and Function	3.58E-03 - 6.23E-05	10

Top-five IPA results for the 162 up-regulated DEGs (Fold change > 2.0 and FDR < 0.05) identified from Gleason-8 tumor versus Gleason-6 tumor comparisons in old patient group

Top Canonical Pathways	p-value	Overlap
LXR/RXR Activation	1.16E-02	3.3% 4/121
Atherosclerosis Signaling	1.26E-02	3.2% 4/124
Inhibition of Matrix Metalloproteases	3.22E-02	5.1% 2/39
Role of hyperchemokinemia in the Pathogenesis of Influenza	3.86E-02	4.7% 2/43
Granulocyte Adhesion and Diapedesis	4.11E-02	2.2 % 4/179
Top Diseases and Disorders	p-value range	Number of Genes
Cancer	2.86E-02 - 8.42E-08	153
Organismal Injury and Abnormalities	2.86E-02 - 8.42E-08	155
Gastrointestinal Disease	2.30E-02 - 1.96E-06	138
Respiratory Disease	2.20E-02 - 9.45E-05	44
Dermatological Diseases and Conditions	2.15E-02 - 1.26E-04	95
Molecular and Cellular Functions	p-value range	Number of Genes
Cellular Development	2.86E-02 - 2.24E-04	36
Cellular Growth and Proliferation	2.86E-02 - 3.08E-04	25
Cellular Movement	1.44E-02 - 5.11E-04	5
Cell Morphology	2.86E-02 - 8.59E-04	24
Cellular Assembly and Organization	2.86E-02 - 8.59E-04	21
Physiological System Development and Function	p-value range	Number of Genes
Organismal Development	2.86E-02 - 2.32E-04	52
Embryonic Development	2.86E-02 - 2.69E-04	44
Nervous System Development and Function	2.86E-02 - 3.37E-04	42
Tissue Development	2.86E-02 - 3.37E-04	43
Organ Development	2.86E-02 - 4.15E-04	24

Top-five IPA results for the 211 down-regulated DEGs (Fold change > 2.0 and FDR < 0.05) identified from Gleason-8 tumor versus Gleason-6 tumor comparisons in old patient group

Top Canonical Pathways	p-value	Overlap
Axonal Guidance Signaling	1.98E-04	2.9 % 14/487
RhoA Signaling	1.03E-03	4.9 % 6/123
Cellular Effects of Sildenafil (Viagra)	1.43E-03	4.6 % 6/131
Agranulocyte Adhesion and Diapedesis	2.18E-03	3.6 % 7/192
Prostanoid Biosynthesis	2.97E-03	22.2 % 2/9

Top Diseases and Disorders	p-value range	Number of Genes
Cancer	7.76E-03 - 1.56E-16	198
Dermatological Diseases and Conditions	6.27E-03 - 1.56E-16	151
Organismal Injury and Abnormalities	8.13E-03 - 1.56E-16	201
Reproductive System Disease	7.27E-03 - 1.78E-09	143
Gastrointestinal Disease	7.92E-03 - 3.70E-09	181

Molecular and Cellular Functions	p-value range	Number of Genes
Cellular Assembly and Organization	5.27E-03 - 2.34E-09	20
Cellular Development	6.22E-03 - 3.60E-08	38
Cellular Growth and Proliferation	5.27E-03 - 3.60E-08	25
Cellular Movement	7.64E-03 - 6.36E-05	59
Cellular Compromise	1.75E-03 - 8.60E-05	8

Physiological System Development and Function	p-value range	Number of Genes
Skeletal and Muscular Development and Function	7.43E-03 - 2.56E-11	46
Organismal Development	7.43E-03 - 1.97E-08	85
Embryonic Development	7.43E-03 - 3.23E-08	59
Organ Development	7.43E-03 - 3.23E-08	51
Tissue Development	7.43E-03 - 3.23E-08	58

Top-five IPA results for the 298 up-regulated DEGs (Fold change > 1.5 and FDR < 0.05) identified from Gleason-8 tumor versus Gleason-6 tumor comparison in young patient group

Top Canonical Pathways	p-value	Overlap*
Cyclins and Cell Cycle Regulation	9.30E-05	8.6 % 7/81
FXR/RXR Activation	1.37E-03	5.6 % 7/126
Mitotic Roles of Polo-Like Kinase	1.73E-03	7.6 % 5/66
LPS/IL-1 Mediated Inhibition of RXR Function*	2.87E-03	4.0 % 9/224
Apelin Liver Signaling Pathway	4.62E-03	11.5 % 3/26

Top Diseases and Disorders	p-value range	Number of Genes
Cancer	1.30E-02 - 5.89E-15	281
Organismal Injury and Abnormalities	1.31E-02 - 5.89E-15	282
Gastrointestinal Disease	1.31E-02 - 8.89E-09	246
Nutritional Disease	2.26E-03 - 4.48E-08	20
Renal and Urological Disease	7.17E-03 - 1.36E-07	57

Molecular and Cellular Functions	p-value range	Number of Genes
Cell Cycle	1.21E-02 - 1.27E-12	69
Cellular Assembly and Organization	1.11E-02 - 1.27E-12	66
DNA Replication, Recombination, and Repair	1.21E-02 - 1.27E-12	41
Cellular Development	1.21E-02 - 2.15E-05	76

Cellular Growth and Proliferation	1.24E-02 - 2.15E-05	76

Physiological System Development and Function	p-value range	Number of Genes
Embryonic Development	1.24E-02 - 4.28E-06	85
Organismal Development	1.31E-02 - 4.28E-06	90
Organismal Survival	3.43E-03 - 3.23E-05	77
Cardiovascular Development and Function	1.31E-02 - 1.03E-04	24
Connective Tissue Development and Function	1.21E-02 - 4.84E-04	45

*Endotoxin lipopolysaccharide (LPS), a major component of the outer membrane of Gram-negative bacteria, potently stimulates host innate immune response. LPS binds the CD14/TRL4/MD2 receptor complex, which promotes the secretion of pro-inflammatory cytokines (IL-1, TNF) in different cell types, but especially in macrophages.

Top-five IPA results for the 349 down-regulated DEGs (Fold change > 1.5 and FDR < 0.05) identified from Gleason-8 tumor versus Gleason-6 tumor comparison in young patient group

Top Canonical Pathways	p-value	Overlap*
Axonal Guidance Signaling	6.32E-05	4.1 % 20/487
Synaptic Long Term Depression	1.44E-04	5.9 % 11/187
Cellular Effects of Sildenafil (Viagra)	1.82E-04	6.9 % 9/131
Thrombin Signaling	3.77E-04	5.3 % 11/209
Signaling by Rho Family GTPases	3.83E-04	4.9 % 12/244

Top Diseases and Disorders	p-value range	Number of Genes
Cancer	2.32E-03 - 2.57E-20	330
Organismal Injury and Abnormalities	2.34E-03 - 2.57E-20	332
Reproductive System Disease	2.25E-03 - 3.67E-17	243
Gastrointestinal Disease	2.24E-03 - 1.86E-15	299
Dermatological Diseases and Conditions	1.05E-03 - 1.65E-13	223

Molecular and Cellular Functions	p-value range	Number of Genes
Cell-To-Cell Signaling and Interaction	2.13E-03 - 3.41E-05	37
Amino Acid Metabolism	2.02E-03 - 6.28E-05	15
Cell Death and Survival	1.36E-03 - 6.28E-05	9
Small Molecule Biochemistry	2.24E-03 - 6.28E-05	40
Cellular Movement 3.86E-04 - 8.48E-05 8		

Physiological System Development and Function	p-value range	Number of Genes
Cardiovascular System Development and Function	2.01E-03 - 6.74E-08	57
Skeletal and Muscular System Development and Function	2.24E-03 - 1.26E-07	51
Respiratory System Development and Function	2.24E-03 - 4.00E-07	22
Organismal Development	2.31E-03 - 8.44E-07	135
Digestive System Development and Function	2.29E-03 - 2.26E-06	25

Highlighted yellow are immune related pathways, functions or diseases.

Supplementary Table 6. Larger metastasis–associated median difference in gene expression of 36 iPAM genes in young group than in old group from Decipher GRID samples

Gene**	Young group (≤ 55 years, n* = 48, 149)			Old group (> 70 years, n* = 68, 121)		
	Median difference	p ***	^FDR	Median difference	p ***	^FDR
ASPN	0.325	< 0.001	< 0.001	0.070	0.277	0.499
KRT15	-0.159	< 0.001	< 0.001	0.002	0.906	0.909
FAM13C	-0.193	< 0.001	< 0.001	-0.054	0.009	0.065
NR4A1	-0.394	< 0.001	< 0.001	-0.128	0.079	0.246
ANO7	-0.241	< 0.001	< 0.001	-0.114	< 0.001	< 0.001
MYBPC1	-0.431	< 0.001	< 0.001	-0.203	0.021	0.108
NCAPD3	-0.458	< 0.001	< 0.001	-0.154	0.061	0.220
AZGP1	-0.531	< 0.001	< 0.001	-0.257	0.030	0.135
CDC42EP5	-0.197	< 0.001	< 0.001	-0.068	0.146	0.311
ITGBL1	0.103	< 0.001	< 0.001	0.055	0.113	0.291
SFRP4	0.275	0.001	0.003	-0.024	0.675	0.814
TOP2A	0.077	0.001	0.003	0.074	< 0.001	< 0.001
SLC22A3	-0.228	0.002	0.006	-0.134	0.002	0.018
LRRN1	0.145	0.004	0.010	0.032	0.439	0.718
C7	-0.160	0.004	0.010	-0.079	0.043	0.172
DPT	-0.089	0.006	0.014	-0.009	0.731	0.822
CD24	0.197	0.014	0.029	0.100	0.673	0.814
ANTXR1	0.115	0.026	0.055	0.015	0.678	0.814
ATP5EP2	0.221	0.031	0.062	-0.026	0.701	0.814
LBH	0.058	0.035	0.066	0.037	0.166	0.332
CYBA	-0.085	0.043	0.077	-0.035	0.398	0.682
PCA3	-0.410	0.052	0.089	-0.733	0.001	0.012
KIF21A	0.102	0.065	0.104	0.022	0.576	0.768
GLO1	0.245	0.069	0.104	-0.150	0.133	0.311
LTF	-0.167	0.069	0.104	-0.062	0.208	0.394
GNPTAB	0.142	0.087	0.125	0.030	0.557	0.768
GLYATL1	-0.098	0.104	0.144	-0.076	0.082	0.246
STRBP	0.053	0.157	0.202	0.022	0.565	0.768
RNF39	-0.056	0.283	0.351	-0.027	0.546	0.768
GMNN	0.052	0.308	0.370	0.082	0.013	0.078
SLC37A3	0.053	0.326	0.379	0.072	0.107	0.291
FBXL8	-0.036	0.487	0.538	0.008	0.806	0.879
MTDH	-0.031	0.493	0.538	0.032	0.532	0.768
UGDH	0.066	0.526	0.557	-0.015	0.909	0.909
DDIT4	-0.014	0.789	0.812	0.059	0.147	0.311
CCDC6	-0.020	0.878	0.878	-0.018	0.851	0.901

^ Benjamini-Hochberg adjusted p value by the p.adjust function in R stats package;
*number of patients with and without metastasis, respectively;
**genes with red font are immune-related genes;
*** Non-parametric ANOVA (p-values generated by percentile bootstrap), implemented in the "Rallfun-v35" R codes (the pb2gen function) from Dr. Wilcox (4), was used to test median differences in gene expression between patients with and without metastasis in each age group.

References

1. Ding Y, Wu H, Warden C, et al. Gene Expression Differences in Prostate Cancers between Young and Old Men. *PLoS Genet* 2016;12:e1006477.
2. Smyth GK, Michaud J, Scott HS. Use of within-array replicate spots for assessing differential expression in microarray experiments. *Bioinformatics* 2005;21:2067-75.
3. Benjamini Y, Hochberg Y. Controlling the False Discovery Rate - a Practical and Powerful Approach to Multiple Testing. *J R Stat Soc B* 1995;57:289-300.
4. Erho N, Crisan A, Vergara IA, et al. Discovery and validation of a prostate cancer genomic classifier that predicts early metastasis following radical prostatectomy. *PLoS One* 2013;8:e66855.
5. Tibshirani R, Hastie T, Narasimhan B, Chu G. Diagnosis of multiple cancer types by shrunken centroids of gene expression. *Proc Natl Acad Sci U S A* 2002;99:6567-72.
6. Wang S, Zhu J. Improved centroids estimation for the nearest shrunken centroid classifier. *Bioinformatics* 2007;23:972-9.
7. Blagus R, Lusa L. Improved shrunken centroid classifiers for high-dimensional class-imbalanced data. *BMC Bioinformatics* 2013;14:64.
8. Aran D, Hu Z, Butte AJ. xCell: digitally portraying the tissue cellular heterogeneity landscape. *Genome Biol* 2017;18:220.
9. Wilcox R. Introduction to Robust Estimation and Hypothesis Testing. 4th edition. *Academic press*; 2016.
